# Do Cochrane summaries help student midwives understand the findings of Cochrane systematic reviews: the BRIEF randomised trial

**DOI:** 10.1186/s13643-016-0214-8

**Published:** 2016-03-01

**Authors:** Fiona Alderdice, Jenny McNeill, Toby Lasserson, Elaine Beller, Margaret Carroll, Vanora Hundley, Judith Sunderland, Declan Devane, Jane Noyes, Susan Key, Sarah Norris, Janine Wyn-Davies, Mike Clarke

**Affiliations:** School of Nursing and Midwifery, Queen’s University Belfast, Medical Biology Centre, 97 Lisburn Road, Belfast, Northern Ireland BT9 7BL UK; Cochrane Editorial Unit, St Albans House, 57-59 Haymarket, London, SW1Y 4QX UK; Bond University Queensland, Robina, QLD 4229 Australia; School of Nursing, Trinity College Dublin, 24 D`Olier Street, Dublin, Ireland; School of Health and Social Care, Bournemouth University, Royal London House R118, Christchurch Road, Bournemouth, BH1 3LT UK; School of Health Sciences, City University London, Northampton Square, London, EC1V 0HB UK; Nursing & Midwifery Studies, Aras Moyola, National University of Ireland Galway, Galway, Ireland; School of Social Sciences, Bangor University, Bangor, LL57 2DG Gwynedd UK; School of Nursing and Midwifery and Social Care, Faculty of Health Sciences and Medicine, Edinburgh Napier University, Sighthill Court, Edinburgh, EH11 4BN UK; Department of Interprofessional Health Studies, College of Human and Health Sciences, Swansea University, Swansea, SA2 8PP UK; Faculty of Health Sport and Science, University of South Wales, Pontypridd, South Wales CF3 71DL UK; School of Medicine, Dentistry and Biomedical Sciences, Centre for Public Health, Queen’s University Belfast, Belfast, Northern Ireland UK

**Keywords:** Randomised trial, Evidence summaries, Structured abstracts, Format, Interpretation, Conclusions, Systematic reviews

## Abstract

**Background:**

Abstracts and plain language summaries (PLS) are often the first, and sometimes the only, point of contact between readers and systematic reviews. It is important to identify how these summaries are used and to know the impact of different elements, including the authors’ conclusions. The trial aims to assess whether (a) the abstract or the PLS of a Cochrane Review is a better aid for midwifery students in assessing the evidence, (b) inclusion of authors’ conclusions helps them and (c) there is an interaction between the type of summary and the presence or absence of the conclusions.

**Methods:**

Eight hundred thirteen midwifery students from nine universities in the UK and Ireland were recruited to this 2 × 2 factorial trial (abstract versus PLS, conclusions versus no conclusions). They were randomly allocated to one of four groups and asked to recall knowledge after reading one of four summary formats of two Cochrane Reviews, one with clear findings and one with uncertain findings. The primary outcome was the proportion of students who identified the appropriate statement to describe the main findings of the two reviews as assessed by an expert panel.

**Results:**

There was no statistically significant difference in correct response between the abstract and PLS groups in the clear finding example (abstract, 59.6 %; PLS, 64.2 %; risk difference 4.6 %; CI −0.2 to 11.3) or the uncertain finding example (42.7 %, 39.3 %, −3.4 %, −10.1 to 3.4). There was no significant difference between the conclusion and no conclusion groups in the example with clear findings (conclusions, 63.3 %; no conclusions, 60.5 %; 2.8 %; −3.9 to 9.5), but there was a significant difference in the example with uncertain findings (44.7 %; 37.3 %; 7.3 %; 0.6 to 14.1, *p* = 0.03). PLS without conclusions in the uncertain finding review had the lowest proportion of correct responses (32.5 %). Prior knowledge and belief predicted student response to the clear finding review, while years of midwifery education predicted response to the uncertain finding review.

**Conclusions:**

Abstracts with and without conclusions generated similar student responses. PLS with conclusions gave similar results to abstracts with and without conclusions. Removing the conclusions from a PLS with uncertain findings led to more problems with interpretation.

**Electronic supplementary material:**

The online version of this article (doi:10.1186/s13643-016-0214-8) contains supplementary material, which is available to authorized users.

## Background

Although systematic reviews are widely accepted as a reliable source of evidence for health care, there are many challenges in overcoming barriers to their use in healthcare decision-making [1, 2]. Related to this, systematic reviews often have to summarise findings from large and complex fields of research. The sheer volume of research alone requires good evidence summary versions to ensure that the key messages are communicated effectively and efficiently to the reader [3]. The *Cochrane Library* provides a collection of full-text systematic reviews developed using rigorous reporting standards and methods [4]. Each review has a plain language summary and a structured abstract, which includes a section for the authors’ conclusions. Significant efforts have been made to improve accessibility and readability of Cochrane Reviews to a broad group of readers including practitioners, policymakers and healthcare consumers [5–7].

A key aspect of improving access to knowledge is to ensure not only that the content of the resource is appropriate but also that the format in which it is presented is fit for purpose. A range of evidence summary formats now exist that build on the recognised need for quick and easy access to evidence and have been categorised by Haynes into a pyramid of six ‘S’s [8]. Structured abstracts are acknowledged widely as the preferred summary format in health professional journals [9] and were developed to assist readers in retrieving, selecting and critically appraising relevant literature at a glance [10, 11]. Plain language summaries (PLS) have evolved to summarise Cochrane Reviews in non-technical language that will be read and understood by a broad readership, including consumers of health care. As with abstracts, PLS will often be read as stand-alone documents [12]. They are increasingly used as a key means of disseminating evidence to different audiences including healthcare practitioners, consumers, journalists, policymakers and researchers [5]. Because evidence summaries such as abstracts and PLS are often the first, and sometimes the only, point of contact for research studies and systematic reviews, it important to identify how helpful they are to potential users, such as health professionals in training and practice.

Consideration has been given to how to communicate the benefits and harms of treatment [13] and on how we best present evidence [7, 14]. For example, which evidence summary format, if any, is most open to erroneous interpretation needs to be considered. Recent studies have shown variability in the interpretation of results [7, 15] and have highlighted the importance of authors’ conclusions in interpreting summary statements. Lai et al. [16] conducted a cross-sectional study with hospital practitioners and medical students in which participants were shown four Cochrane Reviews without the authors’ conclusions. The findings demonstrated that the majority of participants could not generate appropriate conclusions from these review abstracts, in the absence of the authors’ conclusions.

It is also important to explore the impact of other influencing factors, such as prior knowledge, prior belief and complexity of the data being presented. Only 47 % of participants in Lai et al.’s study reported changing inappropriate prior belief about the intervention to something appropriate after reading the systematic review abstract [16]. Research in cognitive psychology suggests that we have many cognitive biases, i.e. thinking patterns based on observations and generalisations that can impact on our judgement [17, 18].

Different professional groups have expressed varying degrees of confidence with using evidence resources to inform their practice. For example , Lai et al. [19] found that doctors were more confident and had a more positive perception of evidence-based practice (EBP) than nurses and allied health professionals. However, confidence and self-assessed knowledge is not a proxy for skills [20]. Professional education provides an early and important opportunity to embed the core skills and concepts of EBP into all aspects of healthcare training. While EBP is widely taught in higher education, concerns have been expressed as to how much the student is learning and how they are applying the knowledge to their broader understanding of policy and practice [21].

The aim of this randomised trial was to see how far the interpretation of systematic review summary formats by midwifery students agreed with expert consensus. The objectives were to determine whether there was a difference in interpretation between the use of structured scientific abstracts and non-technical PLS and, secondly, to determine whether the presentation of authors’ conclusions was needed in order for the primary outcome of the reviews to be correctly interpreted.

## Methods

### Study design

A randomised trial was conducted using a 2 × 2 factorial design (abstract versus PLS, conclusions versus no conclusions). Evidence summaries were randomly allocated to participants using block randomization. The trial was administered via a paper survey to midwifery students at a set time before or after a lecture. A complete set of questionnaires were collated, placed in random order (in blocks of 12) and sequentially numbered before being distributed to the nine participating universities. This ensured that each student was randomly allocated to complete a questionnaire which included one of the four summary formats (abstracts versus plain language summary, with or without conclusions) for each of two selected Cochrane Reviews (one with clear findings and the other with uncertain findings).

The data collection process and questionnaire content were piloted with 118 students at Queen’s University Belfast as part of a survey exploring use and attitudes towards evidence resources, including the *Cochrane Library*. As part of that survey, we asked them to read abstracts or PLS of Cochrane Reviews and we assessed their answers about these reviews. We excluded these pilot survey participants from the main Belfast Reviews of Interventions: Evidence Format trial (BRIEF trial) to avoid unblinding any aspect of the conduct or results of the main trial.

The students were asked to complete the first three sections (student details, use of evidence resources and prior knowledge of the topics of the Cochrane Review that were being studied) of the questionnaire before commencing Section 4, which contained the abstracts or PLS of the two Cochrane Reviews. The students were asked to indicate which of the six statements (shown below) best represented the findings of each review.

### Participants and setting

Midwifery students attending one of nine universities in the UK and Ireland with recognised midwifery training programmes were recruited to the trial, between January 2013 and February 2014. Midwifery students registered for 18-month pre-registration course, 3- or 4-year pre-registration course, post-registration and postgraduate modules were eligible to participate in the study. It was possible that some of the lectures would have other health professionals in attendance in addition to midwives, but the questionnaire was constructed to identify any participants who were not midwives and they were excluded from the analysis.

### Ethics, consent and permissions

Ethical approval was obtained from the School of Nursing and Midwifery Ethics Committee at Queen’s University Belfast (and local approval to conduct the study was also obtained from the ethics committee of each participating nursing department (Bangor University, Bournemouth University, Edinburgh University, National University of Ireland Galway, Swansea University, Trinity College Dublin, University of South Wales). The protocol as approved by the ethics committee can be found in the Additional file 1. The collaborator at each site co-ordinated the consent and questionnaire distribution in a classroom setting.

### Randomisation

Based on the approximate number of eligible students, the random number function in Microsoft Excel was used to place the numbers 1 to 1000 in random order using random blocks of 12 for the four evidence summary formats: format A (abstracts with conclusion), format B (abstracts without conclusions), format C (plain language summaries with conclusions) and format D (plain language summaries without conclusions). Random allocation was in blocks in order to keep the sizes of the groups similar, and the blocks of 12 were used to preserve unpredictability as smaller groups sizes were not anticipated. The random number tables were generated by MC. The questionnaires were ordered in accordance with this random sequence (by FA) and then given a sequential number to ensure distribution in the appropriate sequence. A record which linked the random order to the sequence number was kept centrally so the questionnaires could be tracked. Each centre provided details on the maximum number of students available to participate, and a batch of questionnaires was then sent starting with the next available sequential number.

The students were emailed or given an information leaflet about the study during the week before the questionnaire was administered. On the day of data collection, all participants were advised again that participation was voluntary and that all answers were anonymous. Written consent was obtained from each student, and then the summary formats were presented to the students in the paper questionnaire. Questionnaires were distributed in order from the top of the collated pile of randomised questionnaires, working consecutively along each row of the students in the class. The questionnaire number was for randomisation only and was not linked to student consent or any other data. The students were asked to complete the questionnaires independently, and questionnaire completion was supervised by the researcher or class teacher at each site.

### Study interventions

Students were randomised to receive one of the four evidence summary formats for two Cochrane Reviews in their questionnaire. These Cochrane Reviews had been purposively selected by the study team, one where the effects of the intervention were conclusive (from here on referred to as clear finding reviews) and one where the effects of the intervention were uncertain (as assessed by an expert panel). We considered the strength of the conclusions and the direction and size of effects as reported in the results to determine which reviews to use and identified a shortlist of 10 Cochrane Reviews covering topics of interest to midwives, with abstracts and PLS with similar levels of content. Two reviews related to maternity care were identified through student response to the pilot survey, discussion and consensus of the core study team [22, 23]. Each of these reviews provided similar concluding statements in the abstracts and PLS. The reviews also provided numbers of trials, numbers of women and summary statements of the main findings in both summary formats. The main differences in these examples were the lack of structure in the PLS in comparison to the abstract, no methodological information in the PLS and the reporting of relative risks and confidence intervals for a number of outcomes in the abstract. GRADE statements or summary of findings tables were not presented with either format. The core study team also agreed on the key finding for each review, based on the information in the abstract and PLS, which we regarded as the appropriate response (primary outcome) for the selected reviews. The team also created five other responses for each review so that students could be presented with multiple choices as outlined below. The questionnaires used for each intervention group can be found at http://www.qub.ac.uk/schools/SchoolofNursingandMidwifery/Research/ResearchInPractice/Projects/BriefTrial/.

### Primary outcome measure

The primary outcome measure for BRIEF was the proportion of students who identified the appropriate response to the review summary. The response categories were piloted and refined with a group of midwifery students to ensure clarity and meaningfulness of each category.

Responses to the summary were given using the following categories, which are based on the responses used by Lai et al. [16]:

A. In general, [the intervention] is clearly beneficial.
B. In general, [the intervention] is clearly not beneficial.
C. In general, [the intervention] appears to be beneficial from limited evidence, more studies are needed to confirm the findings.
D. In general, [the intervention] appears to be non-beneficial from limited evidence, more studies are needed to confirm the findings.
E. There is insufficient evidence to comment on whether [the intervention] is, or is not, beneficial.
F. I do not understand the results presented.

Identification of the appropriate response to act as the reference standard was based on a validation exercise conducted with a panel comprising four members of the core study team who are experienced reviewers and two independent experienced reviewers. The panel aimed to achieve consensus about the benefits and harms of the interventions as relayed in the abstracts and PLS and to arrive at an agreed appropriate response from the options in the questionnaire. The panel members were allocated one of the two sets of summary versions to assess. Each member highlighted the key text used to arrive at their response, and their decisions were based solely on the information provided. Additional comments were also noted and, where there was a lack of agreement, the panel discussed the different responses and arrived at a consensus response.

### Sample size calculation

As good data were not available on which to base the sample size calculation, we chose the value that would require the maximum sample size, i.e. that the lowest proportion of students that would identify the appropriate response would be 50 % for any of the types of summary. Then, with 80 % power, we could detect an absolute improvement of 10 % or more, with a sample size of 400 in each group. That is, we would be able to detect a statistically significant odds ratio of 1.50 or above for providing the appropriate answer in either of the main comparisons (abstract versus PLS, conclusions versus no conclusions) with a total of 800 participants in the study.

### Analysis

The primary outcome was the participant’s answer to the question about the main result of the review. This was dichotomised as ‘appropriate’ and ‘other’, with a sensitivity analysis that further divided ‘other’ into ‘near miss’ and ‘incorrect’ (see below).

Logistic regression was used for the primary analysis of the trial, based on the primary outcome of whether or not each participant selected the appropriate response to the review summary. First, the main effects of each participant’s randomised group were fitted (i.e. abstract versus PLS provided, and conclusions versus no conclusions provided). This method was then repeated, adjusting for potential confounders for choosing the appropriate answer, including (i) prior knowledge of the review, (ii) prior belief in the worth of the treatment, (iii) age group (< 20, 20–29, 30–39, 40+ years), (iv) course year, (v) type of midwifery course and (vi) university.

Sensitivity analyses were performed in two ways, firstly, categorising the primary outcome to ‘appropriate’, ‘near miss’ and ‘other’ and secondly, categorising it as ‘appropriate’, ‘near miss’, ‘incorrect’ and ‘other’. Ordinal logistic regression was used for these analyses.

## Results

**Fig. 1:**
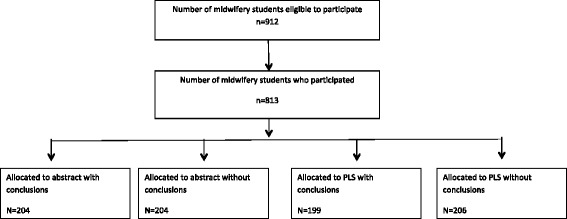
Participant flow diagram

Eight hundred thirteen midwifery students participated in the trial, representing 89 % of students who were enrolled on a midwifery course and therefore eligible to participate in the nine universities. Specific reasons for non-participation were not documented, and we do not have additional information on the students who did not take part. Figure 1 shows the flow diagram of the midwifery students who participated in the trial. The vast majority of the students were women, between 20 and 29 years old, and 74 % were attending a 3-year pre-registration midwifery degree course. There was a good spread of participants across the 3 years of these courses. Two universities had a 4-year course, reflected in a small number of fourth-year students. Participant characteristics were similar across the four intervention groups (Table 1).

**Table 1:** Characteristics of participants

Characteristic	Abstract with conclusions *n* = 204	Abstract without conclusion *n* = 204	PLS with conclusions *n* = 199	PLS without conclusions *n* = 206
% female	99.5	98.5	100	100
Age *N* (%)				
<20	26 (12)	23 (11)	25 (11)	18 (9)
20–29	109 (53)	110 (54)	114 (57)	109 (53)
30–39	47 (23)	46 (23)	43 (22)	59 (28)
40+	21 (10)	22 (11)	15 (7)	19 (9)
Missing	1 (1)	3 (1)	2 (1)	1 (1)
Year of study *N* (%)				
1	80 (39)	81 (39)	82 (41)	85 (41)
2	57 (28)	64 (32)	57 (29)	62 (30)
3	59 (29)	53 (26)	53 (27)	51 (25)
4	8 (4)	6 (3)	6 (3)	8 (4)
Type of course *N* (%)				
Pre-registration 3 year	153 (75)	152 (75)	145 (73)	150 (73)
Pre-registration 4 year	23 (11)	20 (10)	23 (12)	22 (11)
Pre-registration 18 months	15 (7)	18 (9)	18 (9)	18 (9)
Post-registration course	13 (6)	16 (8)	17 (9)	17 (8)

For the clear finding example, 59.6 % of those in the abstract group chose the appropriate response compared with 64.2 % of those in the PLS group (OR 0.82, 95 % CI 0.62 to 1.09). This gives a risk difference (RD) of 4.6 % (95 % CI −0.2 to 11.3; *p* = 0.17) in favour of the PLS (Table 2). Of those in the conclusion group, 63.3 % chose the appropriate answer for this review, compared with 60.5 % of those in the group without conclusions (OR 1.13, 95 % CI 0.41 to 1.50; RD 2.8 %, CI −3.9 to 9.5; *p* = 0.41), which is in favour of provision of conclusions.

**Table 2:** Proportion of participants with the appropriate response for the main results of the review, by randomised comparison

	Number (%) with appropriate response for clear findings review		Number (%) with appropriate response for uncertain findings review	
Comparison 1: abstract versus plain language summary				
	Yes	No	Yes	No
Abstract group	243 (59.6 %)	165 (40.4 %)	174 (42.7 %)	234 (57.4 %)
Plain language group	260 (64.2 %)	145 (35.8 %)	159 (39.3 %)	246 (60.7 %)
Comparison 2: conclusions versus no conclusions				
Conclusions	255 (63.3 %)	148 (36.7 %)	180 (44.7 %)	223 (55.3 %)
No conclusions	248 (60.5 %)	162 (39.5 %)	153 (37.3 %)	257 (62.7 %)

For the uncertain finding review, 42.7 % in the abstract group chose the appropriate answer, compared with 39.3 % of those in the PLS group (OR 1.14, 95 % CI 0.87 to 1.52; RD −3.4 %,CI −10.1 to 3.4; *p* = 0.33), which is in favour of the abstract. The difference in response between the conclusion (44.7 %) and no conclusion group (37.3 %) in choosing the appropriate answer for this review was statistically significant (OR 1.35, 95 % CI 1.02 to 1.79; RD 7.3 %, CI 0.6 to 14.1; *p* = 0.03), which is in favour of the group who were provided with conclusions.

Results of the adjusted analyses are shown in Tables 3 and 4. University was not a significant predictor of choosing the appropriate answer and has been omitted from the tables. For the clear finding review, prior knowledge of the review (compared with no prior knowledge) increased the odds of choosing the correct answer (OR 1.54, 95 % CI 1.08 to 2.20, *p* = 0.02), as did being unsure about prior knowledge of the review (OR 1.77, 95 % CI 1.18 to 2.67, *p* = 0.01). On the other hand, prior belief that the treatment was neither beneficial nor harmful (compared with a belief in its efficacy) decreased the odds of choosing the appropriate answer (OR 0.42, 95 % CI 0.22 to 0.81, *p* = 0.01). There was weak evidence that the older age group (i.e. 40 years and over) was more likely to choose the correct answer, but the confidence interval is wide, due to the small number (*n* = 76) in this age group (OR 1.88, 95 % CI 0.92 to 3.83, *p* = 0.08). There was no significant effect of course year or course type.

**Table 3:** Multivariable logistic regression results for the clear finding review

Factor (reference category)	Odds ratio^a^	95 % CI	*p* value
Main factor 1 (plain language summary)			
Abstract group	0.82	0.61 to 1.10	0.19
Main factor 2 (no conclusions)			
Conclusions	1.16	0.86 to 1.56	0.31
Heard of review result prior (no)			
Yes	1.54	1.08 to 2.19	0.02*
Do not know	1.77	1.18 to 2.67	0.01*
Belief in efficacy of intervention (beneficial)			
Harmful	N/A	no participants	
Neither beneficial nor harmful	0.42	0.22 to 0.81	0.01*
Do not know	0.47	0.20 to 1.08	0.07
Age group (< 20 years)			
20–29 years	1.31	0.79 to 2.18	0.30
30–39 years	1.22	0.70 to 2.13	0.48
40+ years	1.88	0.92 to 3.83	0.08
Course year (year 1)			
Year 2	1.15	0.78 to 1.70	0.49
Year 3	1.14	0.76 to 1.71	0.54
Year 4	1.89	0.63 to 5.69	0.26
Midwifery course type (pre-reg 3 years)			
Pre-registration 18mth	0.88	0.50 to 1.52	0.64
Post-registration	0.86	0.45 to 1.63	0.64
Pre-registration 4 years	1.04	0.57 to 1.90	0.89

^a^Odds ratios are for the outcome of choosing the correct answer about the main result of the review

*Statistically significant at the 5 % level

**Table 4:** Multivariable logistic regression results for the uncertain findings review

Factor (reference category)	Odds ratio^a^	95 % CI	*p* value
Main factor 1 (plain language summary)			
Abstract group	1.14	0.85 to 1.53	0.37
Main factor 2 (no conclusions)			
Conclusions	1.43	1.06 to 1.91	0.02*
Heard of review result prior (no)			
Yes	0.97	0.49 to 1.94	0.94
Do not know	0.98	0.61 to 1.58	0.92
Belief in efficacy of intervention (beneficial)			
Harmful	1.03	0.38 to 2.78	0.95
Neither beneficial nor harmful	0.84	0.42 to 1.70	0.64
Do not know	1.16	0.71 to 1.90	0.56
Age group (<20 years)			
20–29 years	1.69	0.98 to 2.93	0.06
30–39 years	1.59	0.88 to 2.88	0.13
40 + years	2.00	0.98 to 4.08	0.06
Course year (year 1)			
Year 2	1.40	0.96 to 2.05	0.08
Year 3	1.77	1.18 to 2.65	0.006*
Year 4	1.89	0.68 to 5.24	0.22
Midwifery course type (pre-reg 3 years)			
Pre-registration 18 months	1.29	0.74 to 2.24	0.37
Post-registration	0.90	0.46 to 1.77	0.76
Pre-registration 4 years	0.65	0.35 to 1.21	0.17

^a^Odds ratios are for the outcome of choosing the correct answer about the main result of the review

*Statistically significant at the 5 % level

For the uncertain finding review, the only significant predictor of choosing the appropriate answer was course year, with a year on year increase in odds ratio with those in year 3 having a significantly higher odds ratio compared with year 1 students (OR 1.77, 95 % CI 1.18 to 2.65, *p* = 0.006 for year 3). There was a weak evidence that students aged less than 20 years were less likely to choose the appropriate answer (Table 4).

All groups struggled with interpreting the evidence. The percentage of students identifying the appropriate response ranged from 32.8 % in the uncertain finding review presented as a PLS without conclusions through to 64.2 % in the clear finding review presented as a PLS with conclusions. The test for interaction between the two main effects was not statistically significant for either review (*p* = 0.39 for clear finding review, *p* = 0.47 for uncertain finding review), with the interaction ratio being 1.00 in both cases, demonstrating neither a synergistic nor antagonistic effect of the two interventions and, thereby, supporting assumptions underpinning use of the 2 × 2 factorial design [24].

After adjustment for confounders, the odds ratios for the main effects changed very little. Sensitivity analyses, using three and four categories of answer, rather than two, did not change the results meaningfully and are not shown.

## Discussion

Overall, the proportion of appropriate answers was low. There was a small effect of having conclusions on identifying the appropriate answer for the uncertain finding review. Prior knowledge and belief predicted the response to the clear finding review but not to the uncertain finding review, and course year predicted the percentage of students giving an appropriate answer to the uncertain finding review. However, these subgroup analyses should be interpreted with caution, due to multiplicity [25].

Other studies report poor understanding of evidence presented in review summaries. Lai et al. [16] found that only 30 % of medical students and hospital practitioners who had to derive their own conclusion for Cochrane abstracts without conclusions chose the most appropriate conclusions, using similar response categories to those used in this study. The participants also performed most poorly when the results were negative (20 %) or there was limited evidence of effect (25 %). A recent trial by Santesso et al. [14] showed that a new Cochrane PLS format was preferred by members of the public compared to the current version, as used in this study. The new format increased overall understanding of participants from 18 % with the current Cochrane PLS format to 53 % with the new format. While this is encouraging, even this proportion is lower than desired and more work is needed to overcome barriers that still exist to understanding the information presented.

The importance of conclusions, particularly when the results are unclear, highlights the careful consideration required to produce judicious statements that will not unduly influence the reader. There is a need for the use of clear language to communicate size and direction of effect and quality of evidence in reporting the results throughout the text of the review. Zhelev et al. [7] conducted a qualitative study with 21 clinicians, researchers and policymakers using Cochrane diagnostic test accuracy reviews and found that readers reported them as largely inaccessible with further problems arising from poor layout and presentation. Based on their findings, Zhelev et al. recommended clearer presentation of definitions, the use of more accessible formats for numerical results, careful wording of conclusions and an emphasis on the limitations of the results. Santesso et al. [14] found that understanding of the effects of the intervention and the quality of evidence was greater when communicated in qualitative statements as well as in numbers and symbols, with more people obtaining the correct answers when using a standardised language and presentation of key data in a table.

In our BRIEF trial, prior knowledge and prior belief predicted student response on the clear finding review but not the uncertain finding review. Current psychological research demonstrates that beliefs and heuristics impact on our decision-making [25]. Heuristics are simple, unconscious procedures that people use in everyday life to help them find adequate, though often imperfect, answers to difficult questions [17]. A number of heuristics and biases may have influenced decision-making in our study. For example, interpretation of evidence may be influenced by anchoring, which is the tendency to rely too heavily on one piece of information when making decisions. This is usually the first piece of information acquired on that subject but could be the concluding statement. Also, there is a tendency to search for, interpret, focus on and remember information in a way that confirms one’s preconceptions (confirmation bias). Cognitive bias may be easier to correct in some contexts rather than others. For example, Lai et al. [16] found participants were more likely to change their beliefs after reading an abstract if the abstract reported findings of benefit compared to when the underlying review showed no benefit. Future research should consider heuristics and cognitive biases, to see what part they play in the process of interpreting and using evidence summaries and how they may be used to improve the reader’s understanding of the evidence being presented.

Our study was conducted with midwifery students, the majority of whom were studying or had studied evidence-based practice as part of their pre-registration training. Reassuringly, educational experience appears to predict a better response when the findings of the review were uncertain, which suggests that current education is of benefit, when faced with challenging information. However, based on this and other studies we have a long way to go to provide summary evidence that enhances the understanding of a large proportion of evidence users. This is a challenge for evidence providers and educationalists alike. Decades of educational research has led to the suggestion that we need a paradigm shift to outcome-based education [26] which is an active, learner-centred and results-oriented approach to learning [27]. This approach would provide an opportunity to reflect on how we are teaching and assessing systematic reviewing, to explore what evidence is being used in teaching practice and how it is being used by teachers and students [21, 28]. An important additional consideration when training health professionals is linking application of evidence to practical assessments which often drives student-based learning. Consideration should be given to how to introduce a higher level of application of appropriate evidence-based practice into practical assessments such as objective structured clinical examinations (OSCEs), virtual clinical simulation and clinical practice with patients [21]. More in-depth qualitative research could provide important insights into our results and provide valuable information on how we can support evidence users to effectively use the wealth of available evidence both in education and practice [7].

BRIEF was a large, randomised trial conducted in nine universities throughout the UK and Ireland. The method of data collection, randomisation process and survey responses were piloted in one university in advance of the main study. We conducted a validation exercise to identify the appropriate response for the two reviews. We did not assume that our expert panel had the correct answer rather we assessed the information presented and identified the appropriate response from the material provided to the student. Eleven percent of the eligible students did not participate, and we do not know if these students were different from those who participated. Despite the sample size, we did not have enough power to adequately explore an interaction between summary format and providing conclusions. Therefore, while no interaction was found, we cannot assess if an interaction was the cause of the poorest performing group (PLS without conclusions). Also, the clear finding review was updated in the later stages of data collection, which may have impacted on the students’ response through recent exposure. However, although this was a high-profile midwifery review, the appropriate response for its overall findings did not change between the version in BRIEF and the updated version.

## Conclusions

In summary, the BRIEF randomised trial evaluated how midwifery students interpreted the findings from Cochrane Reviews when the evidence was presented in two different summary evidence formats, abstract and PLS, with and without the provision of the authors’ conclusion. Abstracts with and without conclusions generated similar student responses. PLS with conclusions were similar to abstracts. Removing the conclusion from a PLS with uncertain findings created more problems with interpretation. Prior knowledge and belief appeared to predict the response to the clear finding review but not the uncertain finding review, and course year appeared to predict the percentage of students giving an appropriate answer to the uncertain finding review. Future research should take into consideration what factors, in addition to format, might influence the interpretation of evidence summaries.

## Additional file

Additional file 1: BRIEF ethics protocol. (PDF 109 kb)

## Abbreviations

BRIEF trial: Belfast Reviews of Interventions: Evidence Format trial
PLS: plain language summary
EBP: evidence-based practice
OSCEs: objective structured clinical examinations
OR: odds ratio
RD: risk difference
95 % CI: 95 % confidence interval
