# Supplementary material for: Do Cochrane summaries help student midwives understand the findings of Cochrane systematic reviews: the BRIEF randomised trial
Source: Syst Rev. 2016 Mar 1;5:40. doi: 10.1186/s13643-016-0214-8 (PMC4774039; doi:10.1186/s13643-016-0214-8)
Supplement: Additional file 1: — BRIEF ethics protocol. (PDF 109 kb) [file 13643_2016_214_MOESM1_ESM.pdf]

The use of evidence resources in midwifery training and practice: The BRIEF Trial
Section 1: Project Team Details

Name Prof Fiona Alderdice (PI)
Address 1 School of Nursing and Midwifery
Address 2 Queen's University Belfast (QUB)
Address 3 Medical Biology Centre
Town Belfast
Post code BT9 5BN
Contact Details f.a.alderdice@qub.ac.uk
Tel: 02890972865

Co Applicants
Elaine Beller, Statistician, Bond University Queensland Australia
Mike Clarke, Chair of Research Methodology, QUB;MRC Hub for Trials Methodology Research, QUB
Toby Lasserson, Cochrane Editorial Unit, London
Jenny McNeill, School of Nursing and Midwifery, Queen's University Belfast QUB

Centre collaborators: Jayne Noyes, Bangor University; Margaret Carroll Trinity College Dublin; Declan Devane, NUI Galway; Susan Key Edinburgh Napier; Vanora Hundley, Bournemouth University; Judith Sunderland, City University London; Sarah Norris, Swansea University; Janine Wyn-Davies, University of South Wales

Section 3 Background

For the last two decades, emphasis has been placed on the need to provide health care that is informed by evidence of its effectiveness, the premise being that this use of evidence-based interventions will lead to better choices and will optimise health outcomes for the service user and maximise use of finite healthcare resources (Bick and Graham, 2010). However, reports across various settings show different levels of receptiveness to Evidence Based Practice (EBP) and significant barriers still exist to the use of evidence resources by health professionals in training and in practice. These barriers include cultural and social factors, poor use of IT support at point of care, time constraints and variations in professional training (Gosling et al, 2003; Lai, Teng and Lee, 2010). Different professional groups have expressed varying degrees of confidence with using evidence resources. For example , Lai, Teng and Lee (2010) found that doctors were more confident and more positive in their perception of EBP than nurses and allied health professionals.

The Royal College of Midwives (RCM) recently launched a Research and Development (R&D) Strategy (2011) which acknowledges the need to improve the quality of midwifery care through the effective, appropriate use of current and relevant knowledge (RCM, 2011).

Setting the scene for this need, a survey in Scotland in the 1990s found that only 27% of of labour ward midwives claimed to use the Cochrane Database of Systematic Reviews on a regular basis (Hillan, McGuire and Cooper, 1998). While much has changed in the intervening years, both in terms of training and access to evidence resources, the more recent survey by Lai, Teng and Lee (2010) highlights a need to clarify the current attitudes, barriers and use of evidence resources by midwives, in order to recognise the aims of the RCM R&D strategy.

Current educational approaches have evolved to maximise the implementation of evidence in practice through resource-based learning. Resource-based learning is not tied to a single learning theory or to any specific pedagogy (Hill & Hannafin, 2001) and involves the effective use of a wide range of print, non-print and human resources for learning. Building on the ethos of resource-based learning, a key aspect of improving access to knowledge is to ensure not only that the content of the resource is appropriate but also that the format in which it is presented is fit for purpose. A range of evidence summary formats now exist that build on the recognised need for quick and easy access to evidence. Structured abstracts are acknowledged widely as the preferred summary format in health professional journals (Hopewell, Eisinga and Clarke, 2008) and were developed to assist readers in retrieving, selecting and critically appraising relevant literature at a glance (Rosenbaum, Glenton and Oxman, 2010). Plain language summaries have evolved to summarize Cochrane Reviews in a straightforward style that can be understood by consumers of health care. As with abstracts, plain language summaries will often be read as stand-alone documents. Because these evidence summaries are often the first (and some cases the only) point of contact for research studies and systematic reviews, it important to identify how they are used by health professionals both in training and practice.

An additional consideration is that of reader interpretation and which evidence summary format, if any, is most open to erroneous interpretation. Recent studies have highlighted variability in the interpretation of results by systematic reviewers (Shrier et al., 2008) and the importance of authors' conclusions in interpreting summary statements. Lai, Teng and Lee (2011) conducted a cross-sectional study with hospital practitioners and medical students in which participants were shown four Cochrane Reviews without the authors' conclusions. Findings demonstrated that the majority of participants could not generate appropriate conclusions from these reviews in the absence of the authors' conclusions. This highlights not only the value placed on authors' conclusions in evidence summaries but the need for accurate presentation of both results and conclusions.

The aim of this study is to explore pre- and post- registration midwifery students' use of evidence resources and to see what data midwives extract accurately from different summary evidence formats. Focus groups will also help identify the needs and views of midwives in relation to the use of evidence in education and practice.

Section 4: Methodology

Primary Objectives:

1. To establish whether the abstract or the plain language summary of a Cochrane Review is a better aid for midwifery students in identifying both the direction and quality of evidence in the review.
2. To establish whether inclusion of conclusions versus no conclusions in the abstract or the plain language summary of a Cochrane Review is a better aid for midwifery students in identifying both the direction and quality of evidence.
3. To determine if there is an interaction between the type of summary, and the presence or absence of conclusions.

Secondary Objectives

4. To describe the attitudes of midwifery students to evidence based practice.
5. To identify the evidence resources that midwifery students access most frequently.
6. To describe the views of midwifery students to different summary formats.
7. To explore in-depth the needs and views of midwifery students to resources that produce short summaries of evidence, (in particular the evidence from systematic reviews).

Methods

Phase 1 RCT

Design: 2 x 2 Factorial design (abstract v PLS, conclusions v no conclusions)

Participants and setting: Midwifery students, including those registered for 18 month pre-registration course, 3 year pre-registation course, post-registration and postgraduate modules, attending one of nine universities in the UK and Ireland with recognised Midwifery training programmes. This should give a potential sample of 800 students. The students will be recruited during January and February 2013.

Randomisation

A maximum of 800 students will be eligible to participate. Based on this maximum, the random number function in Microsoft Excel will be used to place the numbers 1 to 800 in random order using random blocks of 12 for format A (abstracts without conclusion), format B (Plain Language Summaries without conclusions), Format C (abstracts with conclusions) and Format D (Plain Language Summaries with conclusions). The summary formats will be presented to the students in a paper questionnaire. A complete set of 800 questionnaires will be collated, placed in this random order and sequentially numbered to help maintain the random order throughout data collection for each participating centre. The top questionnaire will be selected each time data are collected in each of the classes. The

questionnaire number is for randomisation only and is not linked to student consent or any other data.

Sample size calculation: Assuming the proportion of participants with the appropriate answer in the lowest group is 0.50, then with 80% power, we could detect an improvement to 0.60 (i.e. 10%), if it exists, with a sample size of 400 in each group. That is, a risk ratio of 1.20 for getting the appropriate answer. This applies to both main questions (Abstract vs PLS, Conclusions vs No Conclusions).

Procedure

All midwifery students registered for 18 month pre-registration course, 3 year pre-registration course, post-registration and postgraduate modules will be invited to participate in the study. In QUB only students attending classes not approached in the pilot study will be included. It is possible that some of the modules will have other health professionals in attendance in addition to midwives. While all students attending these interdisciplinary modules will be invited to participate in the study only midwifery students will be included in the primary analysis. The questionnaire has been constructed to identify which students in these modules are not midwives.

Students will be emailed an information leaflet about the study one week before the questionnaire is administered, via the independent gatekeeper at each centre. Teachers will also make the students aware of the study a week in advance. On the day of data collection, they will make them aware that they will have 10 minutes before the researcher arrives to ensure that those who remain are those who wish to participate. The researchers will enter the class room at a time agreed with the class teacher, to administer the questionnaire. All participants will be advised again that participation is voluntary and that all answers will be anonymous. Written consent will be obtained from each student. The questionnaires will have been placed in random order and they should be distributed from the top of the pile for each class, working consecutively along each row of students. This ensures that each student is randomly allocated to complete a questionnaire which includes one of the four summary formats (abstracts versus plain language summary, with or without conclusions) for each of the two selected Cochrane Reviews.

The students are asked to complete Sections 1-3 of the questionnaire before commencing Section 4, which contains the abstracts or plain language summaries. In Section 4, students will be asked for their opinions on the direction and quality of the evidence using the multiple choice question based on that constructed by Lai et al (2010) (see below). Each questionnaire will have a unique identifying number noted on the corner of each page to allow matching of all the sections for data entry.

Study Intervention

Core sections (same for all versions; Objectives 4 &5)

Section 1: Student details

Section 2: Use of evidence resources

Section 3: Opinion of Cochrane Review topics included in the study

Randomised section (Objectives 1-3)
Section 4: Recall of knowledge from one of four Cochrane Review summaries (Abstract with conclusion, abstract without conclusions, PLS with conclusions, PLS without conclusion). Recall of knowledge will be assessed using the following categories:

A. In general, [the intervention] is clearly beneficial.
B. In general, [the intervention] is clearly not beneficial.
C. In general, [the intervention] appears to be beneficial from limited evidence, more studies are needed to confirm the findings.
D. In general, [the intervention] appears to be non-beneficial from limited evidence, more studies are needed to confirm the findings.
E. There is insufficient evidence to comment on whether [the intervention] is, or is not, beneficial.
F. I do not understand the results presented.

A copy of each of the four questionnaires can be found in the ethics application.

Selection of systematic reviews
A shortlist of 10 systematic reviews were identified as covering topics of interest to midwives and having abstracts and plain language summaries with similar levels of content. Two systematic reviews where identified through discussion and consensus of the core study team. The two reviews selected for the main study are:

Hatem M, Sandall J, Devane D, Soltani H, Gates S. Midwife-led versus other models of care for childbearing women. Cochrane Database of Systematic Reviews 2008, Issue 4. Art. No.: CD004667. DOI: 10.1002/14651858.CD004667.pub2.

Churchill D, Beevers GDG, Meher S, Rhodes C. Diuretics for preventing pre-eclampsia. *Cochrane Database of Systematic Reviews* 2007, Issue 1. Art. No.: CD004451. DOI: 10.1002/14651858.CD004451.pub2.

A validation exercise was set up with four core investigators who are experienced reviewers and two independent experienced reviewers to identify a reference standard for each review summary. The panel aimed to achieve consensus about the benefits and harms of the interventions as relayed in abstracts and PLS from Cochrane Reviews and to arrive at an agreed appropriate response using the options A to F given in the questionnaire. Panel members were allocated one of two sets of summary versions to assess. Each member highlighted the key text used to arrive at their response and their decisions were based solely on the information provided. Additional comments were also noted and, where there was a lack of agreement, the panel discussed the different responses and arrived at a consensus response.

Primary Outcome Measure

The proportion of students who agree with the expert panel's 'appropriate' response identified through the validation exercise.

Analysis

The data from the questionnaire will be entered into SPSS Version 18 and descriptive statistics with significance testing will form the basis of the analysis. The primary objectives will be analysed using a chi-square test of proportions. This will be done for the primary objectives 1 and 2:– Abstract vs PLS, and Conclusions vs No Conclusions. Further analysis will compare the 4 groups, and test for the interaction between summary type and presence/absence of conclusions.

Secondary analyses will include:

1) The primary outcome will be analysed in 3 categories:
   - 'Appropriate' answer
   - One step away from the "appropriate" answer *(*i.e. differs in either direction or quality of response)
   - Other answer.
2) Logistic regression analysis will be conducted to determine whether the 'appropriate' answer is predicted by programme of study, gender, age, years of study, the 3 questions about use of evidence, value placed on research evidence, awareness of the review results prior (adjusted for group allocation).

Phase 2 Focus Groups (Objective 6 & 7)

It is proposed that six focus groups will be conducted with midwifery students; one at each participating centre. Participants for these focus groups will be recruited via email by the gatekeeper at each centre, with the invite letter and information leaflet attached. Those who would like to participate will be asked to respond directly to the researcher and the first 10 volunteers in each group will be invited to participate. The focus groups will last approximately one hour and will be recorded and transcribed. It is anticipated that focus groups will add depth to the survey data. The two Cochrane Reviews used in the focus groups (influenza vaccination and the use of Tamiflu) are both inconclusive in regard to findings and present the evidence with different approaches to content. This will facilitate a better understanding of what student midwives need and want in relation to the presentation of evidence and issues around interpretation of evidence. Focus group questions and the reviews to be discussed can be found in the ethics application. If any comments during discussion are made that might be perceived to constitute unsafe practice this will be dealt with by the project team and the participant will be made aware that they will need guidance on how to manage this in practice. RCM guidance on vaccination and Tamiflu will be available to all participants and all participants will be given a summary of current local guidance on completion of the focus group (Appendix 2).

Analysis

Throughout the focus group interviews the research team will take notes and the focus groups will be recorded digitally. The notes will not identify individual participants by their name. The interviews will be transcribed and anonymised for the analysis of focus group data. The data will be analysed using a broad interpretive qualitative approach based on the principles of thematic analysis.

References

Bick D, Graham ID (2010) The importance of assessing outcomes of evidence based practice. In: Bick, D., Graham, I.D. (Eds.), Evaluating the Impact of Evidence-Based Practice. STTI/Wiley-Blackwell Science (Chapter 1).

Gosling AS, Westbrook JI, Coiera EW (2003) Variations in the use of online clinical evidence: a qualitative analysis. International Journal of Medical Informatics 69, 1-16

Hill J R, Hannafin M (2001) The resurgence of resource-based learning. Educational Technology, Research and Development 49(3), 37-52

Hillan EM, McGuire MM, Cooper M (1998) Computers in midwifery practice: a view from the labour ward. Journal of Advanced Nursing 27 (1), 24–29

Hopewell S, Eisinga A, Clarke M (2008). Better reporting of randomized trials in biomedical journal and conference abstracts. Journal of Information Science 34, 162-173

Lai NM, Teng CL, Lee ML (2010) The place and barriers of evidence based practice: knowledge and perceptions of medical, nursing and allied health practitioners in Malaysia. BMC Research Notes 3, 279 (http://www.biomedcentral.com/1756-0500/3/279)

Lai NM, Teng CL and Lee ML (2011) Interpreting systematic reviews: are we ready to make our won conclusions? A cross-sectional study. BMC Medicine 9, 30 (http://www.biomedicalcentral.com/1741-7015/9/30)

Rosenbaum SE, Glenton C, Oxman AD (2010) Summary-of-findings tables in Cochrane reviews improved understanding and rapid retrieval of key information. Journal of Clinical Epidemiology 63, 620-626

Royal College of Midwives (2011) The Royal College of Midwives Research and Development Action Plan Royal College of Midwives: London

Shrier I, Boivin JF, Platt RW, Steele RJ, Brophy JM, Carnevale F et al (2008) The interpretation of systematic reviews with met-analyses: an objective or subjective process? BMC Medical Informatics and Decision Making 8, 19 (doi:10.1186/1472-6947-9-19)
